# Expression of anti-fungal peptide, β-defensin 118 in oral fibroblasts induced by *C. albicans* β-glucan-containing particles

**DOI:** 10.1590/1678-7757-2021-0321

**Published:** 2022-04-29

**Authors:** Miyuki Sakuma, Kouji Ohta, Shohei Fukada, Hiroki Kato, Takako Naruse, Takayuki Nakagawa, Hideo Shigeishi, Hiromi Nishi, Masaaki Takechi

**Affiliations:** 1 Hiroshima University Graduate School of Biomedical and Health Sciences Department of Oral and Maxillofacial Surgery Hiroshima Japan Hiroshima University, Graduate School of Biomedical and Health Sciences, Department of Oral and Maxillofacial Surgery, Hiroshima, Japan.; 2 Hiroshima University Graduate School of Biomedical and Health Sciences, Program of Oral Health Sciences Department of Public Oral Health Hiroshima Japan Hiroshima University, Graduate School of Biomedical and Health Sciences, Program of Oral Health Sciences, Department of Public Oral Health, Hiroshima, Japan.; 3 Hiroshima University Hospital Department of General Dentistry Hiroshima Japan Hiroshima University Hospital, Department of General Dentistry, Hiroshima, Japan.

**Keywords:** Candida albicans, β-glucan-containing particles, DEFB118, oral fibroblasts

## Abstract

**Objective:**

Although oral fibroblasts are thought to have the potential to enhance host defenses against *Candida albicans* , it is unknown whether they are able to recognize Candida cell components to increase the expression of antifungal peptides, such as defensin factors, against *Candida* infection.

**Methodology:**

We performed expression profiles of defensin genes induced by heat-killed *C. albicans* in oral immortalized fibroblasts (GT1) using cDNA microarray analysis. From those results, quantitative RT-PCR was used to examine the effects of *Candida* β-glucan-containing particles (β-GPs) on β-Defensin 118 (DEFB 118) expression in oral mucosal cells. Furthermore, the antifungal activities of recombinant DEFB 118 against *C. albicans* and *C. glabrata* were investigated using fungicidal assays.

**Results:**

Microarray analysis showed that DEFB118, β-Defensin 129 (DEFB129), and α-Defensin 1 (DEFA1) genes were induced by heat-killed *C. albicans* and that their mRNA expressions were also significantly increased by live as well as heat-killed *C. albicans* . Next, we focused on DEFB118, and found that GT1, primary fibroblasts, and RT7 (oral immortalized keratinocytes) constitutively expressed DEFB118 mRNA expression in RT-PCR. Furthermore, *C. albicans* β-GPs significantly increased the expression of DEFB118 mRNA in GT1 and primary fibroblasts. Although DEFB118 mRNA expression in RT7 was significantly induced by both live and heat-killed C. albicans, C. albicans β-GPs failed to have an effect on that expression. Finally, recombinant DEFB118 significantly decreased the survival of both strains of *C. albicans* in a dose-dependent manner, whereas no effects were seen for both *C. glabrata* strains.

**Conclusion:**

DEFB118, induced by *C. albicans* β-GPs from oral fibroblasts, may play an important role in oral immune responses against *C. albicans* infection.

## Introduction

Oral candidiasis is the most frequently encountered fungal infection caused by *Candida* species. Notably, *C. albicans* is more prevalent in infections by *Candida* than other species due to its various virulence factors,^1 , 2^ and it has been shown capable of adhering to, invading, and damaging oral epithelia during oral infections.^3 , 4 , 5 , 6 , 7^ On the other hand, oral keratinocytes can play a major part in host defenses against *Candida* infection by producing antimicrobial peptides and pro-inflammatory cytokines.^8 , 9 , 10 , 11^ Additionally, oral fibroblasts comprise an abundant cellular component of connective tissue in oral mucosa.^12^ When *C. albicans* invades oral epithelia and continues to the basal epithelial layer, the infection can also reach adjacent connective tissue.^13^ Thus, oral fibroblasts in connective tissues are also required to participate in host defenses against *C. albicans* .

Defensins are diverse members of a large family of antimicrobial peptides that contribute to mucosal host defenses in various organs, and can be classified into α- and β-defensins.^14^ Human α-defensins are expressed in neutrophils, whereas human β-defensins (HBDs) are mainly produced in various epithelial tissues, including those in the skin, lung, kidneys, pancreas, and nasal oral mucosa. In the oral cavity, HBD1, 2, and 3 are well known and expressed in periodontal tissues.^15 , 16^ Though defensin expression can be induced by proinflammatory cytokines, bacteria, and bacterial components, such as LPS,^17 , 18^ a few studies have reported the induction of mRNA expression of some defensins, including HBD2 and 3, by *C. albicans* in oral keratinocytes.^19 , 20^ On the other hand, there are no known reports regarding expressions of defensin members in oral fibroblasts induced by *C. albicans* .

Immune cells, such as monocytes, macrophages, and dendritic cells, recognize pathogen-associated molecular patterns (PAMPs) on the surface of *C. albicans* and then, activate the innate immune system.^21^ Among the major PAMPs, β-glucan is a critical component of the *Candida* cell wall which is exposed on fungal cell surfaces.^21^ Additionally, it has been reported that oral keratinocytes recognize β-glucan, resulting in the promotion of host immune responses against *Candida* infections.^9 , 22^ Although it remains unknown whether oral fibroblasts recognize *Candida* cell wall components, a previous study showed that CX3CL1, a membrane-bound chemokine in gingival fibroblasts, was induced by heat-killed as well as live *Candida* .^23^ Therefore, gingival fibroblasts may recognize PAMPs such as β-glucan and trigger the host defense system to increase expression of antifungal peptides, including defensins against *Candida* infections.

We hypothesized that oral fibroblasts recognize *C. albicans* cell wall components, resulting in an increased expression of antifungal defensins. In this study, expression profiles of defensin genes, induced by heat-killed *C. albicans* in oral immortalized fibroblasts (GT1), were investigated using cDNA microarray analysis, and we identified three defensins induced by *C. albicans* . In analyses of those defensins, we focused on DEFB118, and examined the effects of *Candida* β-glucan-containing particles (β-GPs) on its expression in GT1, as well as the antifungal activity of recombinant DEFB 118 against *C. albicans* .

## Methodology

### Cell lines

GT1, a human oral fibroblast cell line, was established by transfection of human telomerase reverse transcriptase (hTERT), as previously described,^24^ whereas RT7, an immortalized human oral keratinocyte cell line, was established by transfection of hTERT and the human papillomavirus E7 oncoprotein (E7), as previously described.^25^ GT1 cells were cultured in Dulbecco’s modified Eagle’s medium (Sigma Chemical Co., St. Louis, MO, USA) containing 10% fetal calf serum, 100 U/mL penicillin, and 100 mg/mL streptomycin, whereas RT7 cells were cultured in a keratinocyte growth medium supplemented with human epidermal growth factor, insulin, hydrocortisone, calcium, bovine pituitary extract, and gentamicin sulfate amphotericin B (Lonza, Walkersville, MD). Primary oral fibroblasts established in a previous study were also used.^26^

### Microorganisms and growth conditions

*C. albicans* species IFO1385 and IFM48311, as well as *Candida glabrata* IFM5678 and HCG1 were used in this study. IFM48311 and IFM5678 were obtained from the Chiba University Research Center for Pathogenic Fungi and Microbial Toxicoses, whereas IFO1385 was generously provided by the Department of Bacteriology at the Hiroshima University Graduate School of Biomedical Sciences. *C. glabrata* HCG1 was isolated from patients with oral candidiasis receiving treatment at Hiroshima University Hospital, with informed consent for acquisition obtained according to a protocol approved by the Ethical Committee of Hiroshima University (E-1854). All yeast cells were harvested after growth in a Sabouraud’s broth medium (Becton, Dickinson and Company) at 37°C overnight. *C. albicans* IFO1385 organisms were washed twice with phosphate-buffered saline (PBS) and divided into two samples, with the first sample heat-killed at 60°C for 30 minutes.^27^ Live and heat-killed cells were resuspended in their respective media before assays were performed.

### β-glucan-containing particles

*C. albicans* β-GPs were obtained using a hot alkali and acid method, as previously described.^22 , 28^ Briefly, *C. albicans* IFO1385 was cultured in a Sabouraud’s broth medium (Becton, Dickinson and Company), then washed with PBS and suspended in 1% (wt/v) NaOH at 100°C for 24 hours, after which the supernatant for the alkali-soluble fraction was collected. The insoluble residue was treated with 0.5 M acetic acid at 80°C for 24 hours and subjected to repeated washings with PBS. Then, the pellet was lyophilized and stored at 4°C.

### RNA extraction

In a preliminary experiment, the effects of *C. albicans* on cytotoxicity were examined using a cytotoxicity detection kit (Roche Applied Science, Mannheim, Germany). Neither live (10^5^ CFU/ml) nor heat-killed *C. albicans* (10^8^ CFU/ml) caused a significant increase in lactate dehydrogenase release by GT1 fibroblasts, relative to that from the non-infected control cultures, after 12 hours.^29^ Thus, those concentrations of live and heat-killed *C. albicans* were used in the following examinations. For microarray analysis, GT1 cells were seeded into 10-cm culture plates (Cellstar, Greiner Bio-one) and cultured until reaching 70-80% confluence. Then, after washing the plates with PBS and exchanging the previous medium for an antibiotics-free medium, incubation was continued with heat-killed *C. albicans* (10^8^ CFU/mL) for 12 hours. For the RT-PCR analysis, GT1 cells, primary fibroblasts, and RT7 cells were seeded into 6-well culture plates (Cellstar, Greiner Bio-one) and cultured until reaching 70-80% confluence. Then, after washing with PBS and exchanging the previous medium for an antibiotics-free medium, incubation was continued with heat-killed (10^8^ CFU/mL) or live *C. albicans* (10^5^ CFU/mL), and β-GPs (200 mg/mL) for 12 hours. RNA from those cultures was extracted using an RNAeasy Mini Kit (Qiagen, Hilden, Germany). Single-stranded cDNA for a polymerase chain reaction (PCR) template was synthesized using a First Strand cDNA Synthesis Kit (Amersham Biosciences, Uppsala, Sweden), which was then subjected to microarray analysis, as well as RT-PCR and real-time PCR assays.

### Microarray analysis

Microarray analysis was performed using a NimbleGen MicroArray system containing 24,000 genes from 60-mer oligonucleotides. CDNA was cleaned and labeled in accordance with the NimbleGen Gene Expression Analysis protocol (NimbleGen Systems, Inc., Madison, WI, USA). Following hybridization and washing, array slides were scanned using an Axon GenePix 4000B scanner (Molecular Devices Corporation, Sunnyvale, CA, USA) piloted by the GenePix Pro 6.0 software (Axon). The scanned images were imported using the NimbleScan software (NimbleGen Systems, Inc., Madison, WI, USA). Expression data were normalized using quantile normalization and the robust multichip average (RMA) algorithm, included as part of the NimbleScan software package.

### Quantitative real-time PCR analysis and RT-PCR

Synthesized cDNA was used for quantitative real-time PCR analysis with oligonucleotide primers, as shown in Table 1 . Primers were designed using the Primer3 software package (bioinfo.ut.ee/primer3-0.4.0/). Quantitative PCR analysis was performed using a CFX Connect Real-Time PCR Detection System (BIO-RAD laboratories, Inc.) and a SYBR-Green Master Mix (Applied Biosystems, Carlsbad, CA, USA), with initial denaturation at 95˚C for 2 minutes for 40 cycles (denaturation at 95˚C for 15 seconds, annealing at 60˚C for 60 seconds, elongation at 72˚C for 60 seconds), and final extension at 72˚C for 5 minutes. QPCR analysis was performed using a CFX Connect Real-Time PCR Detection system (Bio-Rad Laboratories, Inc.). Relative quantification of mRNA levels noted for the samples was performed according to User Bulletin #2 (Applied Biosystems; Thermo Fisher Scientific, Inc.), with results shown as the mean ± SD from three independent experiments.

**Table 1:** Heat-killed *C. albicans* -induced defensin genes identified by microarray analysis

Gene bank ID	Gene name	Fold change	Primer
NM004084	α-defensin 1 (DEFA1)	5.51	5’- TCCCAGAAGTGGTTGTTTC -3’
			5’- GCAGAATGCCCAGAGTCTTC -3’
NM080831	βb-defensin 129 (DEFB129)	7.48	5’- GGATCACTGCAATGTGGATG -3’
			5’- CCTGGGGTCATAGTGCTGAT -3’
NM054112	β-defensin 118 (DEFB118)	9.18	5’- GGCTCTTCCTATGCTTGTGC -3’
			5’- ACTCAAGGGTGTGGGAGATG -3’

RT-PCR conditions, with the primers shown in Table 1 , from synthesized cDNAs were as follows: 1 × (95°C, 15 minutes), 40 × (95°C, 2 minutes; 55°C, 30 seconds; 72°C, 1 minute), and 1 × (72°C, 7 minutes). β-actin (primer sequence: forward 5’- TCACCCACACTGTGCCCATCTACGA-3’, reverse 5’- CAGCGGAACCGCTCATTGCCAATGG-3’) and DEFB118, as shown in Table 1 , were included as internal controls. Products (DEFB118, 205bp, β-actin; 295 bp) were analyzed by 2% agarose gels containing ethidium bromide in a Tris-acetate-EDTA (TAE) buffer.

### Fungicidal assays

A previously reported colony-forming unit method was used with minor modifications.23,27,30 Overnight cultures of *C. albicans* IFO1385 and IFM48311, and *C. glabrata* IFM5678 and HCG1 were harvested, washed with PBS, and suspended in 10 mM of sodium phosphate buffer (NaPi; pH 6.8). Each *Candida* suspension was diluted to 10^7^ cells/mL^-1^ with NaPi. Then, 10 ml of the *Candida* suspension were inoculated into 200 ml of NaPi, including the appropriate dilution (0, 3, 6, 12, 25 mg/mL) of recombinant DEFA1 (Peprotech, NJ, USA), DEFB129 (CUSABIO, Houston, TX, USA), DEFB118 (Prospec, Ness-Ziona, Israel) or amphotericin B (FUJIFILM Wako Pure Chemical Corporation, Osaka, Japan) as a positive control antifungal drug, then incubated for 2 hours at 37°C. Thereafter, the reaction mixture was diluted with NaPi, plated in Sabouraud agar (Becton, Dickinson and Company), and incubated at 37°C for 48 hours. The total number of *Candida* colonies in each plate was counted, and the percentage of surviving *Candida* , compared to total colony numbers in the control plates ( *Candida* with Napi as indicated, 0 mg/mL), was determined.

### Statistical analysis

Data were analyzed using the Student’s *t* -test and one-way analysis of variance, followed by Dunnett’s multiple comparison test. Values obtained are shown as the mean ± standard deviation.

## Results

### Identification of defensin gene expression induced by heat-killed *C. albicans*

**Figure 1:**
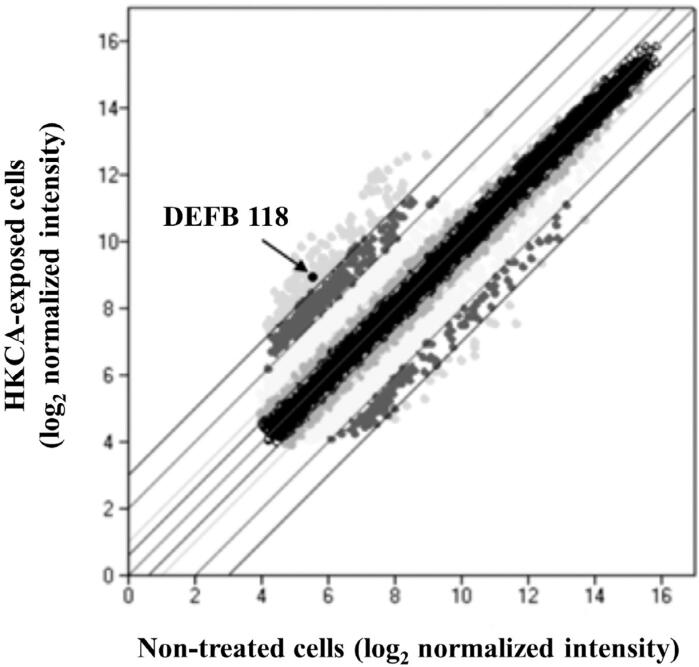
Scatter plot analysis showed several genes that were either up- or down-regulated by heat-killed *C. albicans* (HKCa) in GT1. Cells were incubated with HKCa (108/mL) for 12 hours, and then, gene expression profiling was performed using a cDNA microarray. Upper circles show genes including DEFB118 in cells exposed to HKCA that were up-regulated more than eight-fold as compared to non-treated cells

**Figure 2:**
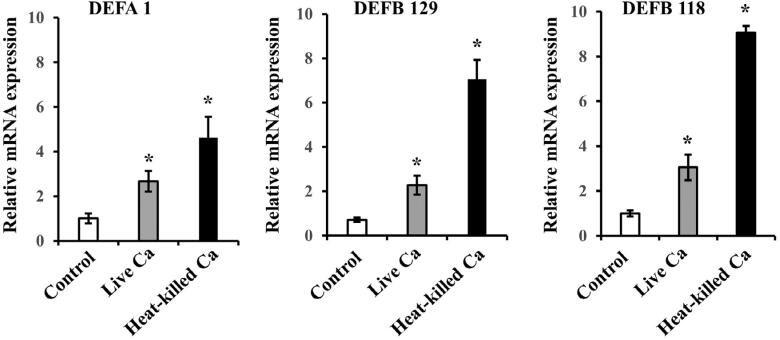
Expression of defensin genes identified by microarray analysis following exposure of GT1 to live and heat-killed *C. albicans* (Ca). Cells were incubated with live Ca (105/mL) or heat killed Ca (108/mL) for 12 hours. Gene mRNA levels are shown as relative to β-actin. Values are shown as fold increase as compared to non-treated cells and as the mean ± SD of three independent experiments. *Significantly different from non-treated cells (paired t-test, P<0.05)

A scatter plot analysis of microarray signals was performed in this study ( Figure 1 ). Among the 24,000 genes analyzed using cDNA microarray results, 17 of the 40 defensin genes were found to be up-regulated more than two-fold in heat-killed *C. albicans* -exposed cells as compared to non-treated cells. Initially, three defensin genes with the highest levels of changed expression induced by heat-killed *C. albicans* , in comparison with non-treated cells, were selected, i.e., β-Defensin 118 (DEFB118), β-Defensin 118 (DEFB129), and α-Defensin 1 (DEFA1) ( Table 1 ). We confirmed mRNA expression of those three defensin genes in GT1, when exposed to live or heat-killed *C. albicans* , using quantitative RT-PCR analysis ( Figure 2 ). Those results showed that mRNA expression of all three defensin genes in GT1 was significantly increased by both live and heat-killed *C. albicans.* Next, in preliminary experiments performed to determine whether those recombinant defensins show antifungal activity against *C. albicans* , the antifungal effects of their proteins were examined using a fungicidal assay. Amphotericin B was used as a positive antifungal control drug. Recombinant DEFB129 and DEFA1 failed to show antifungal activity against *C. albicans* (Supplemental Figure 1 ), whereas DEFB118 did, with the following details shown. As a result, DEFB118 received focus in this study.

### DEFB118 expression in oral fibroblasts induced by *C. albicans* β-glucan-containing particles

**Figure 3:**
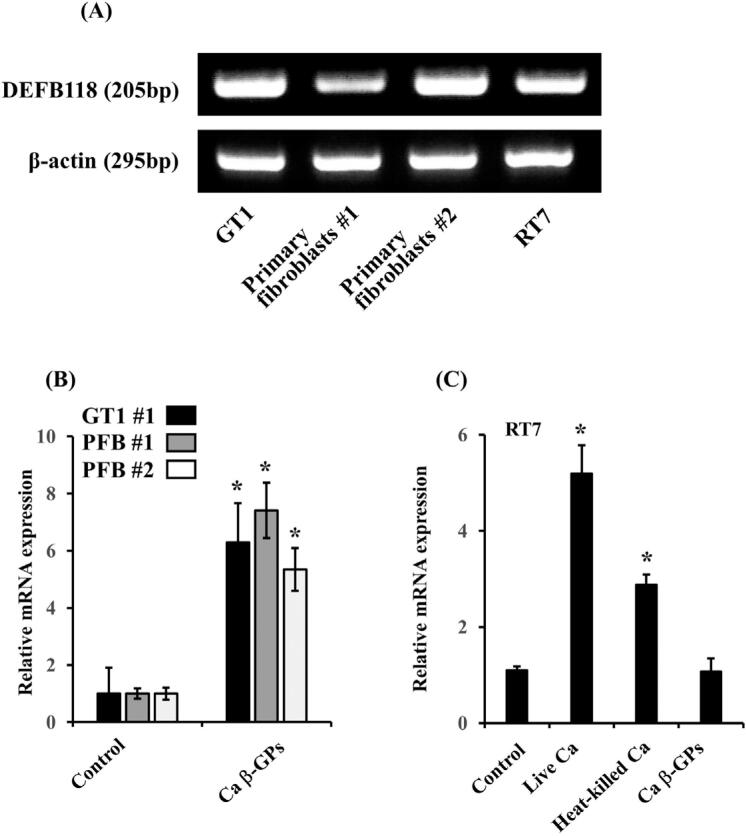
DEFB118 expression in oral fibroblasts and oral keratinocytes. (A) mRNA expression of DEFB118 in GT1, primary oral fibroblasts #1 and #2, and RT7. Total RNA was isolated from each cell line after culturing to confluence. Then, RT-PCR assays of DEFB118 and β-actin were performed. (B) Effects of Ca β-GPs on DEFB118 mRNA expression in primary oral fibroblasts and GT1. GT1 and primary oral fibroblasts (PFB) #1 and #2 were incubated with Ca β-GPs (200 mg/mL) for 12 hours. Gene mRNA levels are shown as relative to β-actin. Values are shown as fold increase as compared with non-treated cells and as the mean ± SD of three independent experiments. *Significantly different from non-treated control (paired t-test, p<0.05). (C) Effects of Ca and Ca β-GPs on DEFB118 mRNA expression in RT7. RT7 were incubated with live (105/mL) or heat killed Ca (108/mL) for 12 hours. Gene mRNA levels are shown as relative to β-actin. Values are shown as fold increase as compared to non-treated cells and as the mean ± SD of three independent experiments. *Significantly different from non-treated cells (paired t-test, p<0.05)

To determine whether oral mucosal cells constitutively express DEFB118, we examined the mRNA expression of DEFB118 in GT1, primary oral fibroblasts, and oral keratinocytes, and RT7 in RT-PCR analysis. Results showed that those oral fibroblasts and keratinocytes constitutively expressed DEFB118 mRNA ( Figure 3A ). Our previous study, using immunofluorescence microscopy, showed that the *Candida* cell wall components β-glucan was present on the cell surfaces of heat-killed *C. albicans* .^22^ Therefore, we examined the effect of *C. albicans* β-glucan-containing particles (β-GPs) on DEFB118 mRNA expression, and those results showed that β-GPs significantly increased DEFB118 mRNA expression in GT1 and two primary oral fibroblasts ( Figure 3B ). Regarding DEFB118 mRNA expression in oral keratinocytes, RT7 was significantly increased by both live and heat-killed *C. albicans* , β-GPs failed to affect DEFB118 mRNA expression in RT7 ( Figure 3C ).

### Antifungal activity of DEFB118

**Figure 4:**
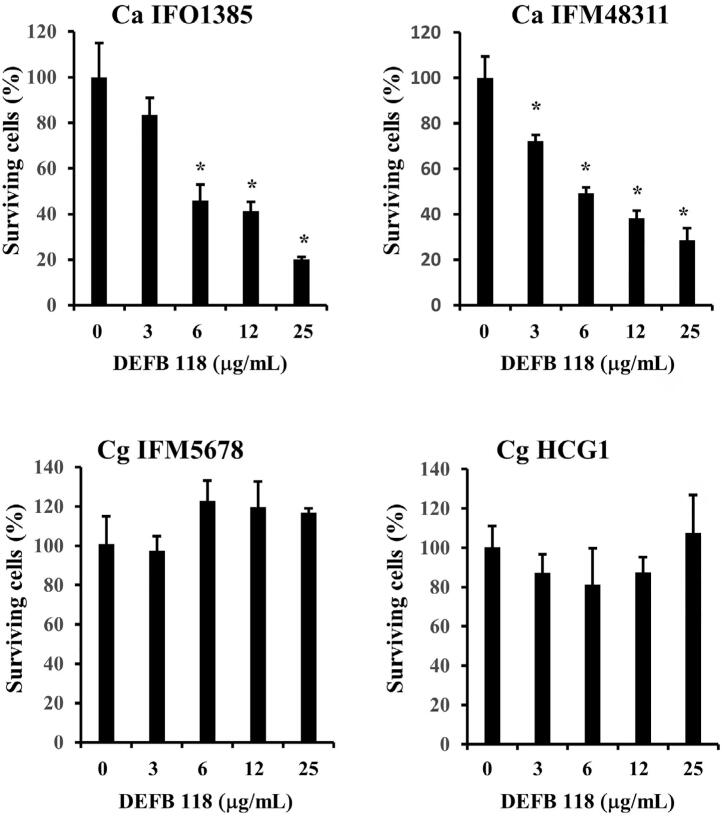
Antifungal activity of recombinant DEFB118. Overnight cultures of C. albicans IFO1385 and IFM48311, and *C. glabrata* IFM5678 and HCG1 were harvested, washed, and suspended in NaPi. Each *Candida* suspension was inoculated in NaPi with the indicated dilution of recombinant DEFB118 and incubated for two hours at 37°C. The mixtures were then separately plated in agar and incubated at 37°C for 48 hours. The total number of *Candida* colonies in each plate was counted, then, the percentage of surviving *Candida* as compared to total colony numbers in the control plates ( *Candida* with Napi as indicated, 0 mg/mL), was determined. Values are shown as the mean ± SD of three independent experiments. *Significantly different from 0 mg/mL (Dunnett’s test, p<0.05)

To investigate whether DEFB118 showed antifungal activity against *Candida* , the fungicidal effects of recombinant DEFB118 on *C. albicans* and *C. glabrata* species were examined. Recombinant DEFB118 significantly decreased the survival of both strains of *C. albicans* in a dose-dependent manner, whereas it failed to affect either of the two *C. glabrata* strains ( Figure 4 ).

## Discussion

Previous studies have found that oral fibroblasts show a host immune response against *C. albicans* .^23 , 29 , 31^ Dongari-Bagtzoglou, et al. reported that IL-8 and IL-6 protein production was secreted from human oral fibroblasts exposed to live *C. albicans* , whereas the *C. albicans* fixed by paraformaldehyde had little effect on their production.^29^ In our previous investigation, the effects of *C. albicans* on the expression of 12 different chemokines in oral keratinocytes and fibroblasts were examined.^23^ Results showed that, while live *C. albicans* increased the level of expression of multiple chemokines in oral keratinocytes, nearly no effects on their expressions were noted in oral fibroblasts. However, among the chemokines examined, we found that CX3CL1 in oral fibroblasts was dramatically increased by live, as well as heat-killed, *C. albicans* . In this study, both live and heat-killed *C. albicans* induced the expression of the anti-microbial peptides DEFB118, 129, and DEFA1. Together, these results suggest that oral fibroblasts recognize *Candida* cell wall components as PAMPs, activating host immune response against *C. albicans* infection.

These results also indicated that the mRNA expressions of three defensin genes present in GT1 cells, DEFA1, DEFB129, and DEFB118, were induced by both live and heat-killed *C. albicans.* DEFA1 is also known as human α-defensin 1 (HNP-1), which has been found in microbicidal neutrophils, as well as in the gingival crevicular fluid of patients with aggressive and chronic periodontitis, as compared with a control patient group.^32^ DEFB 129 and 118 were initially reported by Kao, et al. They performed a genome-wide search using ORFeome-based peptide databases of airway epithelial cells, which showed six novel β-defensins, including those.^33^ DEFB129 mRNA is known to be expressed in human testis specimens, whereas Xie, et al. found that they are highly expressed in various segments of the epididymis in pigs,^34^ and another study showed that porcine DEFB129 protein decreased bacterial endotoxin-induced inflammation and apoptosis in intestinal epithelial cells.^35^ The DEFB 118 gene is located on chromosome 20q11, and its mRNA expression can be detected in pancreas and testis specimens. DEFB 118 protein, which consists of a conserved β-defensin-specific cysteine core and a long anionic C-terminal domain, is the only anionic b-defensin in the 20q cluster.^32 , 36^ Lin, et al.^37^ (2021) showed that DEFB118 recombinant proteins reduced *E coli* -induced inflammation and intestinal injury in mice. Additionally, DEFB118 was found to significantly decrease cell apoptosis in intestinal porcine epithelial cells exposed to an *E. coli* challenge and downregulate the expression of inflammatory cytokines.^38^ However, there is no known previous report regarding DEFB118 expression in the oral cavity or its antifungal properties. In this study, DEFB118 mRNA expression in oral fibroblasts and keratinocytes was found to be increased by *C. albicans* , and recombinant DEFB118 protein showed antifungal properties against *C. albicans* . Thus, DEFB118 may have an important role in the oral mucosal defense system response against *C. albicans* infection.

β-glucan is a key PAMP that has been detected in tissues with *Candida* infection and shown to activate host immune responses. Although the β-glucan layer in the *Candida* cell wall is initially masked by its outer layers of mannan, β-glucan becomes exposed under various conditions, such as drug treatment.^39^ In our previous study, immunofluorescence microscopy revealed β-glucan on the cell surfaces of heat-killed *C. albicans* , which was also detected on the surface of oral epithelia invaded by *C. albicans* following immunofluorescence staining.^22^ These results showed that *C. albicans* β-GPs increased DEFB 118 mRNA expression in oral fibroblasts. Furthermore, these and previous findings indicate that oral fibroblasts can recognize β-glucan in oral epithelia during *Candida* infection, which promotes the induction of DEFB118 as an antifungal response. Also, we previously found that oral keratinocytes recognized *C. albicans* β-GPs and increased heme oxygenase 1 (HO-1).^22^ However, β-GPs failed to affect DEFB 118 mRNA expression in oral keratinocytes in this examination. Thus, immune responses induced by β-GPs may differ among cell types.

Recognition of β-glucans by Dectin-1, a receptor that binds β-glucans and triggers phagocytosis of fungi in macrophages, mediates a variety of anti- *Candida* responses to produce inflammatory cytokines from various cell types.^39 , 40 , 41^ However, its function in oral fibroblasts is largely unknown. Tamai, et al. found that gingival fibroblasts expressed Dectin-1 on the cell surface, though heat-killed *C. albicans* failed to affect induction of Dectin-1 expression.^42^ In this study, the knockdown of Dectin-1 failed to affect the expression of DEFB 118 mRNA in GT1 (data not shown). Thus, the expression mechanism of β-GP-induced DEFB 118 in oral fibroblasts may differ from that of the immune response activated by the recognition of Dectin-1.

Defensins are known to have a broad-spectrum killing activity toward gram-positive and -negative bacteria,^43^ as well as potency toward some fungi, whereas β-defensins 1-3 were found to have direct fungicidal activity toward *C. albicans* .^43^ Furthermore, recombinant DEFB118 showed antibacterial activity against *E. coli* , an ability to destroy the permeability of the *E. coli* membrane, and change the morphology of the bacterial surface,^44^ though it is unknown whether the DEFB118 protein has antifungal activity. These results showed that DEFB118, but not DEFA1 or DEFB129, has antifungal activity toward *C. albicans* , whereas *C. glabrata* organisms showed resistance to that. Some investigators have reported differences among *Candida* species, including *C. albicans* and *C. glabrata* , in regard to the antifungal activities of antimicrobial peptides. Although HBD2 and HBD3 have been shown to have antifungal activities against *C. albicans* , *C. tropicalis* , *C. parapsilosis* , and *C. krusei* , no antifungal effect was found with some *C. glabrata* strains.^45^ In another study, HBD2 promoted the disruption of the membrane integrity of *C. albicans* , but failed to affect *C. glabrata* .^46^ Additionally, the cationic antifungal peptide Histatin 5 was reported to decrease the growth of *C. albicans* and other *Candida* species, such as *C. kefyr* , *C. krusei* , and *C. parapsilosis* , whereas *C. glabrata* was insensitive to that peptide.^47^ These, along with those previous findings, indicate that *C. glabrata* may possess mechanisms to resist the antifungal activities of antimicrobial peptides such as DEFB118.

## Conclusion

In this study, DEFB118 mRNA expression in oral fibroblasts was shown to be increased by *C. albicans* and its cell wall component β-GPs. Additionally, DEFB118 showed antifungal activity against *C. albicans* . Thus, DEFB118, induced by *C. albicans* β-GPs, from oral fibroblasts may play an important role in oral immune response against *C. albicans* infection.
